# The Edmonton Obesity Staging System and pregnancy outcomes in women with overweight or obesity: A secondary analysis of a randomized controlled trial

**DOI:** 10.1111/cob.12510

**Published:** 2022-02-24

**Authors:** Sarah Louise Killeen, Cara A. Yelverton, Aisling A. Geraghty, Maria A. Kennelly, Shane Eakins, Lily Farrell, Jillian F. Fagan, John Mehegan, Fionnuala M. McAuliffe

**Affiliations:** ^1^ UCD Perinatal Research Centre, School of Medicine University College Dublin, National Maternity Hospital Dublin Ireland; ^2^ School of Agriculture and Food Science University College Dublin Dublin Ireland; ^3^ HRB Centre for Health and Diet Research, School of Public Health, Physiotherapy and Sports Science University College Dublin Dublin Ireland

**Keywords:** birth outcomes, cardiometabolic health, Edmonton Obesity Staging System, nutrition, obesity, pregnancy

## Abstract

The Edmonton Obesity Staging System (EOSS) is a proposed clinical practice tool to determine obesity severity. In a secondary analysis of the Pregnancy Exercise and Research Study (PEARS) (a mobile‐health‐supported lifestyle intervention among pregnant women with body mass index [BMI] ≥25 kg/m^2^), we apply the EOSS and explore relationships with pregnancy outcomes. In early (14–16 weeks) and late (28 weeks) pregnancy, fasting lipids and glucose were measured, blood pressure was extracted from medical records and maternal well‐being was assessed using the WHO‐5 Well‐being Index. Pearson's correlations, chi‐square statistics and multiple logistic regression were used to identify relationships. One‐way analysis of variance was used to compare groups. Pregnant women (*n* = 348) were mean (SD) age 32.44 (4.39) years and median (interquartile range) BMI 28.0 (26.57, 29.88) kg/m^2^. Using metabolic criteria only, 81.9% and 98.9% had raised EOSS scores in early and late pregnancy. From early to late pregnancy, EOSS scores increased by 60.1%. Of these, 10.5% experienced a 2‐point increase, moving from stage 0 to stage 2. There was a potential relationship between early EOSS and large for gestational age (*χ* ^2^ = 6.42, df (2), *p =* .04), although significance was lost when controlled for confounders (*p =* .223) and multiple testing. Most women with BMI ≥25 kg/m^2^ had raised EOSS scores, limiting the clinical utility of the tool.

**What is already known about this subject**

- The Edmonton Obesity Staging System (EOSS) has been proposed as an adjunct to body mass index to assess the severity of overweight or obesity and guide treatment prioritization.
- Outside of pregnancy, higher EOSS scores in people with obesity have been associated with increased risk of adverse outcomes like postoperative complications and mortality after bariatric surgery.
- Pregnancy is associated with physiological changes in cardiometabolic factors but there is a paucity of data on how these changes impact application of the EOSS in pregnancy.

**What this study adds**

- Most women with overweight or obesity present with clinical dyslipidaemia in early pregnancy that increases throughout gestation.
- Increased EOSS scores were mostly driven by total cholesterol above clinical cut‐offs.
- In the current format and without data on advanced obesity‐associated complications, use of the EOSS is likely to be of limited value in the antenatal setting.

## 1 INTRODUCTION

Obesity is recognized by the World Obesity Federation, the World Health Organisation and others as a chronic, progressive, relapsing disease.^1^ Potential obesity can be identified using body mass index (BMI) ≥30 kg/m^2^. The severity of obesity can be described using BMI classes, namely class 1 or low‐risk (30–34.9), class 2 or moderate risk (35–39.9) and class 3 or high‐risk obesity (≥40 kg/m^2^).^2^ Pre‐pregnancy overweight or obesity is common, affecting up to 42% of women in the United States.^3^ In pregnancy, a raised BMI is associated with increased risk of adverse maternal and child outcomes.^4^, ^5^, ^6^ Women with obesity require appropriate support and management to reduce the risk of complications in pregnancy and beyond.^7^ While useful on a population basis, BMI does not provide insight into body composition, an important predictor of health outcomes, or the impact of excess adiposity on markers of health.^8^, ^9^ The American Association of Clinical Endocrinologists recommends the use of complication‐based schema to inform obesity management.^10^, ^11^

The Edmonton Obesity Staging System (EOSS) is intended to provide clinically relevant insight into health‐related risk for those with obesity.^12^ It involves the classification of those with obesity into distinct groups based on their medical, psychological and functional health status. Stage 0 is given when the individual has no signs of obesity‐related risk, stage 1 is given when there are subclinical risk factors and stages 2–4 are given in the presence of established obesity‐related comorbidities. The tool was compared by its developers to the ‘tumour, node, metastasis’ system in oncology medicine.^12^, ^13^ Outside of pregnancy, higher EOSS scores have been associated with increased risk of postoperative complications and mortality after bariatric surgery.^14^ A recent review of the evidence suggests that EOSS scores may better predict health service usage and treatment outcomes compared to BMI.^15^

To date, only one study has been published using the EOSS in a pregnant population. In this study of women attending for induction of labour, the rate of caesarean delivery was higher in women with a BMI ≥25 kg/m^2^ and stage 3 EOSS scores, compared to those in stage 2 and below.^16^ In our study, we are the first to apply stage 0–2 EOSS to a general pregnancy cohort of women with BMI ≥25 kg/m^2^, recruited as part of a randomized controlled trial. The aim of this is to determine the severity and change in EOSS scores in women with overweight or obesity but otherwise healthy pregnancies. As a secondary aim, we explore potential relationships between EOSS and pregnancy outcomes. This will provide valuable information about the potential clinical utility of the scheme in identifying risk during pregnancy.

## 2 MATERIALS AND METHODS

This is a secondary analysis of participants recruited as part of the Pregnancy Exercise and Nutrition Research Study (PEARS) trial which was conducted between March 2013 and August 2016 at the National Maternity Hospital in Dublin, Ireland. The study had institutional ethical approval from the clinical research centre and written maternal consent. The PEARS study (ISRCTN registry, https://www.isrctn.com/ [accessed 1/10/2021], ISRCTN29316280) was a randomized controlled trial of a mobile‐health behavioural lifestyle intervention with smartphone app support to prevent gestational diabetes mellitus (GDM) in a pregnant population with overweight or obesity. Details of the study protocol and results have been published previously.^17^, ^18^ In brief, women were randomized to the intervention using computer‐generated allocations in a ratio of 1:1 for usual care versus intervention. The intervention involved a single education session at the start of the study. This included advice on low glycaemic index diets, delivered by a research dietitian or nutritionist. It also included an exercise prescription of 30 min of physical activity for 5 days a week, given by an obstetrician. This information was re‐enforced through a specifically designed smartphone application, fortnightly emails and two face‐to‐face study visits, all underpinned by behaviour change theory. Women in the control group were managed according to local and national guidelines and the advice they received may have varied in relation to nutrition, physical activity and gestational weight gain.^17^ Women were screened for eligibility at their first antenatal visit by reviewing their patient charts. Women were eligible if they had a BMI between 25.0 and 39.9 kg/m^2^, singleton pregnancy, absence of previous GDM or any other medical illness requiring treatment. The primary outcome was an oral glucose tolerance test to diagnose GDM, according to the International Association of Diabetes in Pregnancy Study Groups criteria at 28–30 weeks' gestation.^17^

### 2.1 Study sample

**FIGURE 1:**
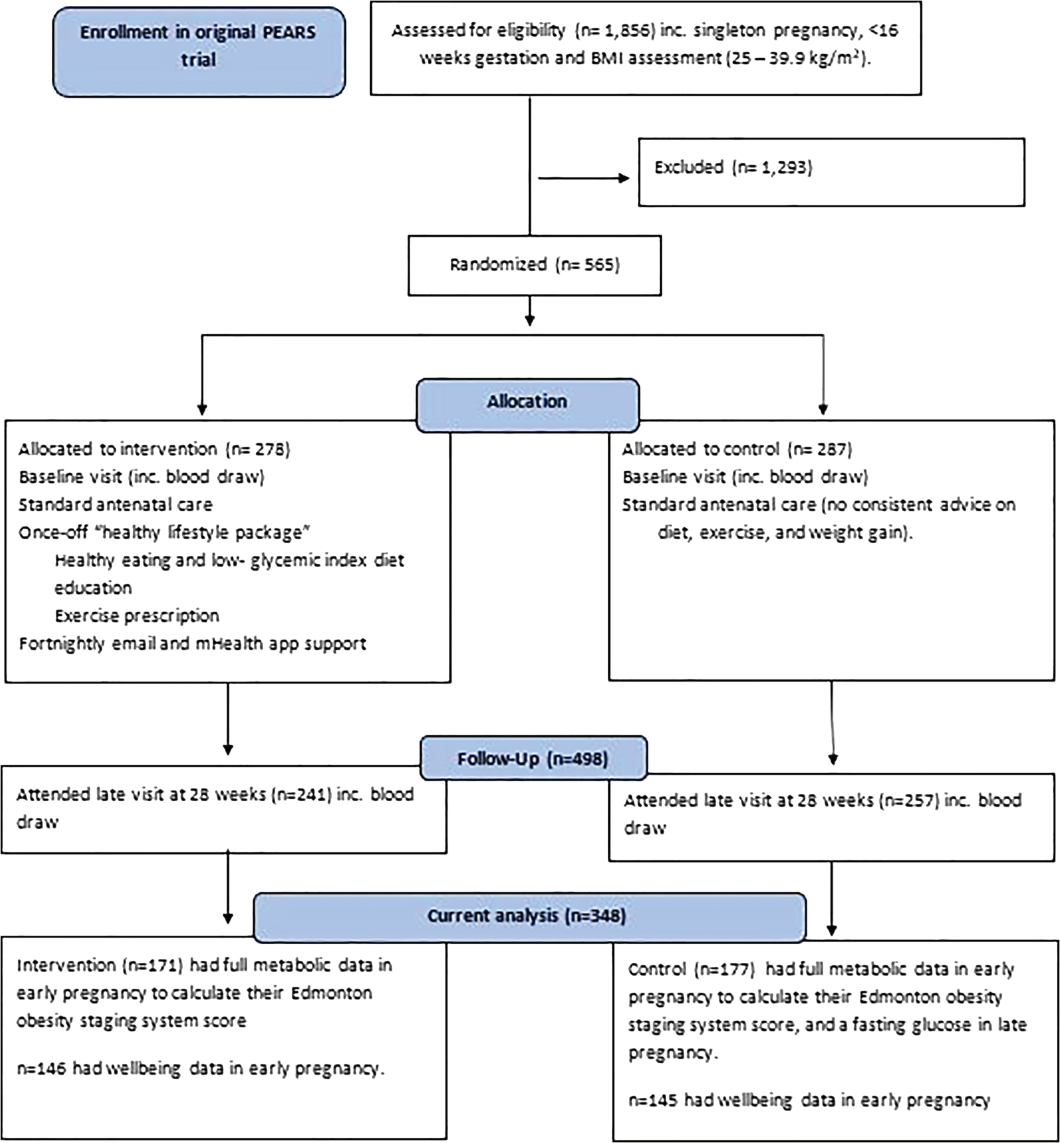
Flow chart of study and details of study sample

This study uses data from the PEARS trial (Figure 1).^18^ A total of 18 (9 in the intervention and 9 in the control) of the 565 women included in the original trial did not attend their first study visit.^18^ Of the remaining women, data were available for baseline blood pressure (*n* = 447), fasting lipids (*n* = 505) and fasting glucose (*n* = 485). The sample in this secondary analysis (*n* = 348) represents those women for whom data were available in early pregnancy on all the cardiometabolic markers needed for EOSS classification (*n* = 350), excluding one twin pregnancy (*n* = 2 mother–child pairs) in the intervention group. More information can be found in Figure 1, adapted from Kennelly et al.^18^

### 2.2 Data collection

#### 2.2.1 Maternal characteristics

All women had their height and weight measured by a healthcare professional at their first antenatal visit, at approximately 10–16 weeks gestation. Maternal weight was measured to the nearest 0.1 kg with the mother in light clothing, using a SECA weighing scales (SECA GmbH & Co. KG, Hamburg, Germany). Maternal height was measured to the nearest 0.1 cm, using a wall‐mounted stadiometer, after removal of footwear. Maternal height and weight were used to calculate BMI. Demographic information collected at the baseline visit included maternal age, ethnicity, parity, and smoking status. Highest level of educational attainment was assessed through a questionnaire at the baseline visit, in which there were six options. The options ranged from ‘completed no schooling’ to ‘completed third level’. Economic advantage was assessed using the Pobal Haase‐Pratschke (HP Pobal) Deprivation Index, a neighbourhood deprivation score based on Irish census data that considers the relative advantage or disadvantage of the mothers' location of residence.^19^, ^20^ Women were classified as ‘above’ or ‘below’ average economic advantage using HP Pobal scores greater than or less than one. A composite measure for socioeconomic status was created based on maternal education (completed third‐level education yes/no) and estimated economic advantage (economic advantage or disadvantage) using the method described by O'Brien et al. A four‐level categorical variable was created ranging from ‘economic disadvantage and did not complete third level’, ‘economic disadvantage and completed third level’, ‘economic advantage and did not complete third level’ and ‘economic advantage and completed third level’.^21^ This variable was further classified into socioeconomically advantaged (yes/no). Women classified as ‘economically disadvantaged and did not complete third level’ were assigned a ‘no’ scoring while all other forms of advantage were classified as ‘yes’.

#### 2.2.2 Cardiometabolic markers

Blood samples were collected at the baseline visit and the study follow‐up (28 weeks) after an overnight fast of at least 8 h. At the shortest possible interval post venepuncture, blood samples were centrifuged at 3000 rpm for 10 min, and the aliquots were stored at −80°C, pending analysis. Glucose was analysed using the AU680 Chemistry analyser (Beckman Coulter Inc., High Wycombe, UK) and the hexokinase method. Total cholesterol, high‐density lipoprotein (HDL) cholesterol and triglyceride concentrations were analysed on a Roche Cobas 702 analyser (Roche Diagnostics). Low‐density lipoprotein (LDL) cholesterol was estimated using the Friedewald equation.^22^ Blood pressure was extracted from antenatal medical records. Average systolic and diastolic values over the early (10–16 weeks gestation) and late (28 weeks) study periods were calculated to allow for EOSS classification.

#### 2.2.3 Maternal well‐being

Maternal well‐being was assessed in early (14–16 weeks) and late (28 weeks) pregnancy using the World Health Organisation (WHO)‐5 Well‐being Index.^23^ Participants were asked to answer five distinct questions on their well‐being in the previous 2 weeks. The questions ask how frequently the participant (1) ‘felt cheerful and in good spirits’, (2) ‘felt calm and relaxed’, (3) ‘felt active and vigorous’, (4) ‘woke up feeling fresh and rested’ and (5) ‘felt their daily life has been filled with things that interest’ them. There were six possible answers on the Likert scale, gaining from 0 to 5 points per question. The highest scoring response is ‘all of the time’ which gains five points, followed in descending order by ‘most of the time’, ‘more than half the time’, ‘less than half the time’, ‘some of the time’ and ‘at no time’. The scores for each of the five questions were added together, resulting in a total score that could range from 0 (lowest possible well‐being) to 25 (highest possible well‐being). The raw scores were multiplied by 4 to create a percentage. A score below <13 suggests reduced well‐being while a score less than <7 suggests potential depression.^24^, ^25^, ^26^

### 2.3 Application of the EOSS

Firstly, cardiometabolic markers were used to classify women into no risk (stage 0), some risk (stage 1) and higher risk (stage 2) (Table 1). Different cut‐offs have been used for the individual cardiometabolic markers in the EOSS across a variety of studies, as detailed in the recent review by Atlantis et al.^15^ In the only study to apply the EOSS in pregnancy to date, women were classified as having an EOSS score of 1 in the presence of subclinical cardiometabolic risk factors associated with obesity, namely borderline hypertension not requiring medical therapy, impaired glucose tolerance or abnormal gestational diabetes screen; however, no specific cut‐offs were provided.^16^ As pregnancy or female‐specific cut‐offs are yet to be established in the context of the EOSS, we selected the biochemical cut‐offs used by Canning et al.^15^, ^27^ The criteria used to apply the EOSS in early pregnancy included fasting glucose, total cholesterol, LDL cholesterol, HDL cholesterol and triglyceride (Table 1). Higher EOSS scores indicate greater metabolic derangement. An EOSS score of 0 was assigned if all the cardiometabolic markers were within the cut‐offs. We did not apply high risk or end‐stage criteria (EOSS stages 3 and 4) to this cohort as the presence of known conditions such as angina pectoris, myocardial infarction, heart failure or type 2 diabetes were exclusion criteria for trial.^27^, ^28^ Women with a well‐being score <13 were given an EOSS score of 1 and those with <7 were given a score of 2 (Table 1). In late pregnancy, the same criteria were used except for fasting glucose as this was not available at 28 weeks because an oral glucose tolerance test was measured at this time instead.^18^ As detailed above, GDM was identified at 28–30 weeks gestation, using the criteria of the International Association of Diabetes in Pregnancy Study after an oral glucose tolerance test.^17^ Pregnancy‐induced hypertension or pre‐eclampsia was identified from medical records. A diagnosis of GDM, pregnancy‐induced hypertension or pre‐eclampsia resulted in a late pregnancy stage 2 EOSS score, as described by Demsky et al.^16^ We did not have data available on obesity‐associated functional complications or other conditions such as kidney disease, so these were not included in the scoring.

**TABLE 1:** Individual criteria used for application of the Edmonton Obesity Staging System (EOSS)

	Stage 0	Stage 1	Stage 2^a^
Glucose (mmol/L)	<5.6	5.6–6.9	>6.9
Triglyceride (mmol/L)	<1.7	1.7–2.26	>2.3
Total cholesterol (mmol/L)	<5.2	5.2–6.1	>6.1
LDL cholesterol (mmol/L)	<3.3	≥3.3	>4.2
HDL cholesterol (mmol/L)	≥1.6	<1.6	<1.0
Systolic blood pressure (mmHg)	<130	130–140	>140
Diastolic blood pressure (mmHg)	<85	85–90	>90
WHO 5 Well‐being score	≥13	<13	<7

*Note*: EOSS stage 1 or 2 is given if the individual has any one or more criteria in line with the corresponding cut‐offs. Early pregnancy EOSS scores used all variables in the table while late pregnancy EOSS included triglyceride concentrations, total cholesterol, LDL cholesterol, HDL cholesterol, systolic blood pressure, diastolic blood pressure and WHO 5 Well‐being score only.

Abbreviations: HDL, high‐density lipoprotein; LDL, low‐density lipoprotein; WHO, World Health Organisation.

^a^ A diagnosis of PIH, PET or GDM also resulted in a stage 2 EOSS score in late pregnancy.

### 2.4 Outcomes

The primary outcome in this study is EOSS score in early and late pregnancy. Secondary outcomes included gestational weight gain, mode of delivery, birthweight, birth length, head circumference and a variety of categorical outcomes including pre‐term delivery (<37 weeks gestation), small for gestational age (SGA) (birthweight <10th centile), large for gestational age (LGA) (birthweight >90th centile), macrosomia (birthweight >4000 g) and low birthweight (<2500 g). Data on neonatal outcomes were retrieved from medical records.

### 2.5 Statistical analysis

Categorical variables are presented as number and frequency (%). Continuous variables were assessed for normality through visual inspection of histograms, the Kolmogorov–Smirnov test for normality, and inspection of descriptive data including the mean, median and skewness. All non‐normally distributed data were log^10^ transformed for regression analysis. Continuous variables are presented as mean (standard deviation) if they are normally distributed or median and interquartile range (25th, 75th centile) for skewed data. Comparison statistics were generated through independent sample *t*‐tests. Chi‐square (*χ* ^2^) tests were used to compare categorical variables. For comparison of categorical variables across EOSS stages, sub‐effect testing was completed post hoc to determine the differences in the 2 × 3 table. Fishers' exact tests were used when expected cell count assumptions were violated. Pearson's correlations were used to assess relationships between variables. Any analysis that was suggestive for a relationship (*p* < .05) was investigated in multiple logistic or linear regression models for categorical or continuous outcomes respectively. The first model was single variable regression followed by two models with a forced entry approach for potential confounders, chosen a priori. The second model included maternal age, maternal baseline BMI (≥30 kg/m^2^ yes/no), and study group (intervention yes/no). The third model additionally included parity (one or more previous pregnancies yes/no), ethnicity (White yes/no), smoking in early pregnancy (current smoker yes/no) and maternal socioeconomic status (composite score based on education and economic advantage). Statistical analysis was performed using IBM Statistical Package for Social Sciences software for Windows, version 26.0 (SPSS Inc., Chicago, IL). All analyses were performed with pairwise deletion of missing variables. We applied the Benjamini‐Hochberg correction for multiple testing with a false discovery rate of 0.20, which is appropriate to support hypothesis generation. At first, *p* values <.05 were considered statistically significant. These values were compared to their corresponding *q* value to determine significance when adjusted for multiple comparisons.

## 3 RESULTS

### 3.1 Maternal characteristics

**FIGURE 2:**
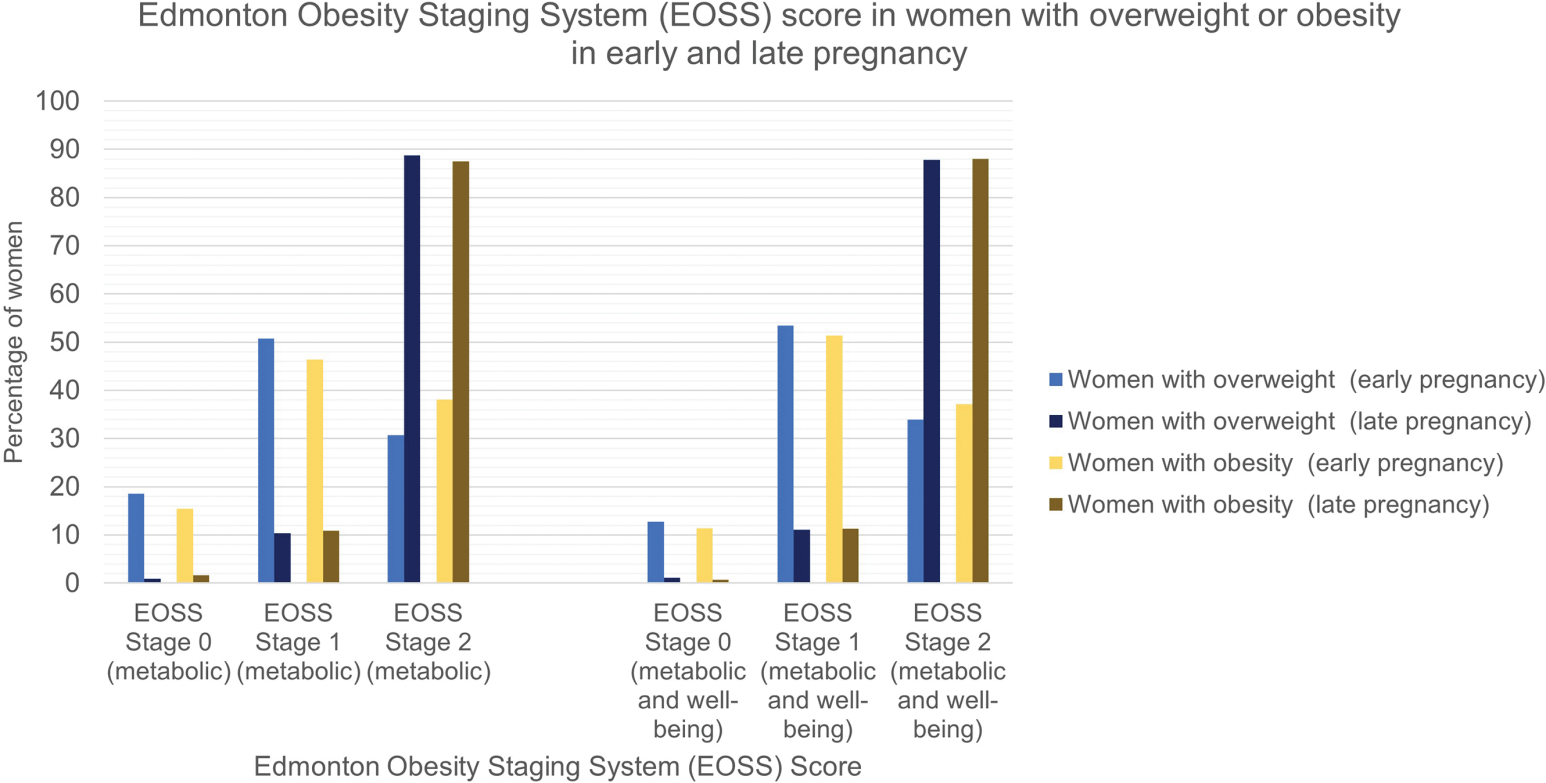
Edmonton Obesity Staging System (EOSS) score in early and late pregnancy by maternal body mass index

Table 2 includes the demographics of the cohort (*n* = 348). Median (interquartile range) BMI was 28.0 (26.57, 29.88) kg/m^2^ and mean (SD) age was 32.44 (4.39) years. Approximately a quarter (24.1%) of the sample had obesity. There was no difference in metabolic EOSS scores in early (*p =* .436) or late (*p =* .907) pregnancy between women with and without obesity. There were also no differences in early (*p* = .877) or late (*p* = .580) pregnancy when well‐being was added to the criteria (Figure 2).

**TABLE 2:** Maternal and infant characteristics in the PEARS study (*n* = 348)

	*n*	Mean (SD)
Age (years)	347	32.4 (4.4)
Body mass index (kg/m^2^)*	348	28.0 (26.6, 29.9)
Body mass index category (*n*, % obesity)	348	84 (24.1)
Ethnicity (*n*, % White)	338	320 (94.7)
Education (*n*, % completed third level)	337	208 (61.7)
Smoking in early pregnancy (*n*, % current)	300	13 (4.3)
Parity (*n*, % 1 or more)	348	163 (46.8)
Socioeconomic status (*n*, % above average advantage)	348	250 (71.8)
Study group (*n*,% intervention)	338	165 (49.4)
Early glycaemic index*	275	58.4 (55.3, 62.0)
Late glycaemic index*	220	57.2 (54.1, 60.2)
Early glycaemic load*	275	129.0 (109.8, 150.6)
Late glycaemic load*	220	121.6 (99.3, 142.6)
Early exercise (Mets)*	301	459.0 (198.0, 787.5)
Late exercise (Mets)*	233	495.0 (198.0, 699.0)
Maternal outcomes		
Gestational diabetes (*n*, %)	317	42 (13.2)
Pre‐eclampsia or pregnancy‐induced hypertension (*n*, %)	314	22 (7.0)
Mode of delivery (*n*, % caesarean delivery)	344	92 (26.7)
Infant outcomes		
Infant sex (% male)	340	84 (24.1)
Birthweight (g)	344	3640.2 (553.5)
Low birthweight (% <2500 g)	344	9 (2.6)
Macrosomia (% >4000 g)	344	78 (22.7)
Small for gestational age (*n*, % <10th centile)	322	24 (7.5)
Large for gestational age (*n*, % >90th centile)	322	35 (10.9)
Placental weight (g)	303	661.1 (143.9)
Birth length (cm)	317	51.2 (2.4)
Head circumference (cm)*	312	35.2 (34.3, 36.0)
Gestational age at delivery (days)*	342	284.0 (275.0, 288.0)
Preterm birth (*n*, % <37 weeks)	342	15 (4.4)

*Note*: Continuous data are presented as mean ± standard deviation (SD) unless ‘*’ which is median (interquartile range). Early refers to data collected between 14 and 16 weeks and late refers to data collected at 28 weeks' gestation.

Abbreviation: Met, metabolic equivalent of task.

### 3.2 Application of the EOSS in early and late pregnancy

Using the metabolic criteria only, 285 (81.9%) had a raised EOSS score (≥1) in early pregnancy, while 273 (98.9%) had an EOSS score ≥1 in late pregnancy. Of these, 113 (32.5%) had stage 2 EOSS scores in early pregnancy, while 244 (88.4%) had an EOSS score of 2 in late pregnancy (see Table 3). In early and late pregnancy, the most common factor resulting in a stage ≥1 score was a total cholesterol level >5.2 mmol/L. The next most common value resulting in raised EOSS classification was a reduced HDL cholesterol. Of those with available data, the majority had a high well‐being score (>13) in early (*n* = 206/291, 70.8%) and late pregnancy (*n* = 1984/223, 82.5%). A well‐being score <7, which is suggestive of potential mental health disorder such as depression, was found in 8 (2.7%) women in early and 6 (2.7%) in late pregnancy.

**TABLE 3:** Edmonton Obesity Staging System in early and late pregnancy

	Early pregnancy		Late pregnancy^a^		
	*n*	*n* (%)	*n*	*n* (%)	
EOSS stage 0 (metabolic only)	348	63 (18.1)	276	3 (1.1)	
EOSS stage 1 (metabolic only)	348	172 (49.4)	276	29 (10.5)	
EOSS stage 2 (metabolic only)	348	113 (32.5)	276	244 (88.4)	
EOSS stage 0 (metabolic and well‐being)	291	36 (12.4)	189	2 (1.1)	
EOSS stage 1 (metabolic and well‐being)	291	154 (52.9)	189	21 (11.1)	
EOSS stage 2 (metabolic and well‐being)	291	101 (34.7)	189	166 (87.8)	
Triglyceride <1.7 mmol/L	348	276 (79.3)	293	85 (29.0)	
Triglyceride 1.7–2.3 mmol/L	348	56 (16.1)	293	114 (38.9)	
Triglyceride >2.3 mmol/L	348	16 (4.6)	293	94 (32.1)	
Total cholesterol <5.2 mmol/L	348	144 (41.4)	293	22 (7.5)	
Total cholesterol 5.2–6.1 mmol/L	348	128 (36.8)	293	54 (18.4)	
Total cholesterol >6.1 mmol/L	348	76 (21.8)	293	217 (74.1)	
LDL cholesterol <3.3 mmol/L	348	195 (55.5)	293	52 (17.7)	
LDL cholesterol 3.3–4.2 mmol/L	348	110 (31.6)	293	75 (25.6)	
LDL cholesterol >4.2 mmol/L	348	45 (12.9)	293	166 (56.7)	
HDL cholesterol >1.6 mmol/L	348	171 (49.1)	293	107 (36.5)	
HDL cholesterol 1–1.6 mmol/L	348	137 (39.4)	293	114 (28.9)	
HDL cholesterol <1 mmol/L	348	40 (11.5)	293	72 (24.6)	
Systolic blood pressure < 130 mmHg	348	335 (96.2)	334	315 (94.3)	
Systolic blood pressure 130–140 mmHg	348	10 (2.9)	334	19 (5.7)	
Systolic blood pressure >140 mmHg	348	3 (0.9)	334	0 (0)	
Diastolic blood pressure <85 mmHg	348	334 (98.9)	334	330 (98.8)	
Diastolic blood pressure 85–90 mmHg	348	4 (1.1)	334	4 (1.2)	
Diastolic blood pressure >90 mmHg	348	0 (0)	334	0 (0)	
Fasting glucose <5.6 mmol/L	348	345 (99.1)		‐
Fasting glucose 5.6–6.3 mmol/L	348	3 (0.9)		‐
Fasting glucose >6.3 mmol/L	348	0 (0)		‐
WHO‐5 Well‐being score >13	291	206 (70.8)	223	184 (82.5)	
WHO‐5 Well‐being score 7–13	291	77 (26.5)	223	33 (14.8)	
WHO‐5 Well‐being score <7	291	8 (2.7)	223	6 (2.7)	

*Note*: Early pregnancy refers to data collected at 14–16 weeks gestation while late pregnancy refers to data collected at 28 weeks. For each parameter, variables are presented in descending order, with criteria for EOSS stage 0 presented first, followed by stage 1, and then stage 2.

Abbreviations: EOSS, Edmonton Obesity Staging System; HDL, high‐density lipoprotein; LDL, low‐density lipoprotein; WHO, World Health Organisation.

^a^ In late pregnancy, stage 2 was given if gestational diabetes, pregnancy‐induced hypertension, or pre‐eclampsia was diagnosed.

### 3.3 Change in EOSS throughout gestation

Out of the 276 women who had metabolic EOSS criteria in early and late pregnancy, two women (0.7%) experienced a reduction in their EOSS score over time. Using the metabolic criteria, over half (*n* = 166, 60.1%) experienced an increase in their EOSS score. Most of these women (*n* = 137, 49.6%) experienced a one‐unit increase while 29 (*n* = 10.5%) women experienced a 2 unit increase in score, moving from stage 0 to stage 2. There were no differences in the change in EOSS metabolic (Fisher's exact statistics 2.06, *p =* .604), or EOSS metabolic and well‐being (Fisher's exact statistics 1.53, *p =* .782) scores between the intervention and control group.

### 3.4 Pregnancy outcomes according to EOSS score

In un‐adjusted, chi‐square testing, data were suggestive of a relationship between early pregnancy metabolic EOSS scores ≥2 and incidence of LGA (*χ* ^2^ = 6.42, df (2), *p =* .04, *q* = 0.01) (Table 4). The relationship between maternal metabolic EOSS scores in early pregnancy and LGA was also seen in unadjusted, singe variable binary logistic regression, where each one‐unit increase in EOSS score was associated with increased odds of delivering an LGA infant (OR = 1.72, 95% CI 1.00, 2.96, *p =* .049). This did not survive adjustment for multiple comparisons (*q* = 0.017). The significance of the relationship between EOSS score and LGA was lost when controlled for confounders (model 2 *p =* .060, model 3 *p =* .223). In model 3, the OR for EOSS stage 1 was 0.82 (95% CI 0.24, 2.80, *p =* .821) and the OR for stage 2 was 1.74 (95% CI 0.51, 5.94, *p =* .374). There were no other statistically significant differences in pregnancy outcomes based on EOSS scores in early pregnancy, using either the metabolic only (all *p* values >.05) (Table 4) or metabolic and well‐being values (all *p* values >.05). In late pregnancy, there were also no additional statistically significant relationships with pregnancy outcomes, using both criteria (all *p* values >.05).

**TABLE 4:** Maternal and foetal outcomes by metabolic Edmonton Obesity Staging System (EOSS) score

	Early pregnancy (metabolic only)							Late pregnancy (metabolic only)						
	EOSS 0		EOSS 1		EOSS ≥2		*p* value	EOSS 0		EOSS 1		EOSS ≥2		*p* value
	*n*		*n*		*n*			*n*		*n*		*n*		
Gestational age at delivery (days)*	59	284.0 (275.0, 289.0)	170	284.0 (275.0, 289.0)	113	283.0 (274.0, 287.0)	.966	3	269.0 (266.0, 269.0)	29	285.0 (276.0, 289.50)	242	284.0 (275.0, 288.0)	.082
Birthweight (g)	60	3558.12 (623.57)	171	3652.89 (522.29)	113	3664.56 (561.21)	.444	3	3778.33 (452.94)	29	3586.72 (504.70)	243	3656.41 (563.59)	.936
Low birthweight (<2500 g)	3	5.0	4	2.3	2	1.8	.534	0	0.0	1	3.4	6	2.5	.584
Macrosomia (>4000 g)	10	16.7	41	24.0	27	23.9	.471	1	33.1	3	10.3	64	26.3	.159
SGA (<10th centile)	6	10.7	11	6.8	7	6.7	.592	0	0.0	1	3.4	18	7.5	.762
LGA (>90th centile)	5	8.9	12	7.5	18	17.1	.040	1	33.3	1	3.4	28	11.6	.191
Placental weight (g)	50	636.72 (131.37)	151	655.07 (136.54)	102	681.98 (158.31)	.146	3	631.0 (37.32)	28	642.46 (156.77)	219	663.72 (144.06)	.891
Birth length (cm)	53	50.91 (3.11)	158	51.21 (2.24)	106	51.46 (51.75)	.418	3	50.0 (1.32)	28	51.12 (2.07)	219	51.23 (2.52)	.581
Head circumference (cm)*	53	35.0 (34.0, 36.0)	155	35.30 (34.30, 36.20)	104	35.0 (34.50, 36.18)	.442	3	35.0 (34.70, 35.0)	26	35.35 (34.03, 36.0)	219	35.20 (34.50, 36.30)	.689
Mode of delivery (caesarean delivery)	15	25.0	40	23.4	37	32.7	.207	1	33.3	7	24.1	62	25.2	1.000
Preterm birth (<37 weeks)	3	5.1	10	5.9	2	1.8	.252	0	0.0	1	3.4	10	4.1	.924

*Note*: Continuous data are presented as mean (standard deviation) unless * which is median (interquartile range of 25th, 75th centile). Categorical variables (low birthweight, macrosomia, small for gestational age, large for gestational age, mode of delivery and preterm birth) are reported as *n* (%) of the outcome. *p* values for continuous variables are generated through analysis of variance while categorical variables were compared using chi‐square statistics, except in the case of cell numbers below expected count, in which Fisher's exact statistics are reported.

Abbreviations: LGA, large for gestational age; SGA, small for gestational age.

As a sub‐analysis, the incidence of LGA births was compared in women with raised EOSS scores ≥1 (*n* = 30, 11.3%) versus EOSS scores of zero (*n* = 5, 8.8%) and no statistically significant difference was seen (*χ* ^2^ = 0.32, df (1), *p =* .575). When women with EOSS stage 2 were compared to women with stage ≤1, the proportion of women delivering an LGA baby was significantly higher for women with high risk (stage 2 metabolic) EOSS in early pregnancy (*n* = 18, 17.1%) versus those with some or no risk (stage ≤1) (*n* = 17, 7.8%), *χ* ^2^ = 6.330, df (1), *p =* .012. The significance of this finding was lost when controlled for multiple comparisons (*q* = 0.003). Using a categorical variable of EOSS score ≥2, the odds of delivering an LGA infant increased when controlled for model 2 confounders (maternal age, study group and BMI) (OR = 2.39, 95% CI 1.15, 4.95 *p =* .019) but significance was lost when controlled for all confounders (model 3) including ethnicity, parity, socioeconomic status and smoking (OR = 2.03, 95% CI 0.90, 4.55, *p =* .087). There was no statistically significant difference in the proportion of women delivering an LGA infant across metabolic EOSS groups in late pregnancy (Table 4). The relationship between early EOSS score and LGA was not significant when well‐being was included (Fisher's exact statistics = 4.23, *p =* .114). Grouping women based on early EOSS metabolic and well‐being stage 2 (*n* = 15, 16.0%) or below (*n* = 15, 8.5%) was also not statistically significant (*χ* ^2^ = 3.49, df (1), *p =* .062).

## 4 DISCUSSION

### 4.1 Main findings

Most women (81.9%) had adverse cardiometabolic profiles in early pregnancy, resulting in EOSS scores ≥1. Over half (60.1%) experienced an increase in their EOSS scores throughout gestation and 10.5% moved from EOSS stage 0 to 2. In late pregnancy, 98.9% of women had a score ≥1. Raised EOSS scores were driven mostly by cholesterol. Adding well‐being to the scoring criteria categorized a greater number of women with higher EOSS in early pregnancy (87.6%) but the proportion of women with raised scores in late pregnancy was similar (98.9%). There was no impact of the PEARs intervention on EOSS values. In early pregnancy, data were suggestive of a relationship between EOSS and LGA, but significance was lost when controlled for all confounders and adjusted for multiple comparisons. On sub‐analysis, the potential relationship between EOSS and LGA appeared due to EOSS stage 2 scores; however, this relationship also did not survive adjustment for multiple comparisons. No relationships with pregnancy or birth outcomes were found for late pregnancy, using any criteria.

### 4.2 Interpretation

Outside of pregnancy, the EOSS has shown some promise in predicting adverse outcomes such as increased mortality and risk of postoperative complications after bariatric surgery.^14^ Using data from individuals with a BMI ≥25 kg/m^2^ in the National Health and Human Nutrition Examination Surveys up to 2006, those with EOSS stage 2 had higher mortality compared to those with 0 or 1.^13^ The impact of higher EOSS scores was also seen in the study by Kuk et al., in which they found an increased risk of mortality in data from 29 000 participants in the Aerobics Center Longitudinal Study, with EOSS stage 2 but not stage 1 obesity.^29^ In some studies, the predictive value appears to be greater with increasing EOSS stages (i.e. stages 3 and 4). These are applied when the individual has advanced obesity‐associated conditions such as established organ damage, significant psychopathology or functional impairments.^14^ The stronger associations between late EOSS stages and adverse outcomes may be due to the high prevalence of metabolic derangement in people with obesity, limiting the difference in individuals when comparing lower stages. Kuk et al. found that out of 54 089 men and women with a BMI ≥30 kg/m^2^, only 5.8% had healthy cardiometabolic values using their cut‐offs.^30^

In our study, unadjusted analyses were suggestive of a relationship between raised EOSS scores and LGA. Sub‐analysis suggested that this was driven by stage 2, but significance was lost when controlled for multiple testing. Categorization of women with raised EOSS scores was mostly driven by cholesterol. A recent observational study of over 500 pregnant women found an association between maternal lipids and LGA, independent of BMI and GDM.^31^ Only one other study has explored the role of EOSS in pregnancy. Demsky et al. applied the EOSS to 345 women attending for induction of labour. They found the overall rate of caesarean delivery was 35.8%, 29.9%, 43.2%, and 90.5% for women assigned an EOSS category 0, 1, 2, and 3, respectively.^16^ We did not find an association with caesarean delivery, but like our study, their data suggest the predictive value of EOSS is most evident at later EOSS stages. In the absence of data on advanced obesity‐associated co‐morbidities to allow for stage 3 or 4 classification, our study suggests EOSS stage 0–2 may not be useful in predicting outcome due to the large proportions of women classified as stage 1 or 2.

Pétursdóttir Maack et al. assessed the predictive value of metabolically healthy versus unhealthy categorization on the risk of complications in 2849 women with overweight or obesity in pregnancy.^32^ Criteria included systolic blood pressure >130 mmHg; diastolic blood pressure >85 mmHg, random glucose >6.8 mmol/L or apolipoprotein B/apolipoprotein A1 (apo B/apo A1) ratio >0.8.^32^ The risk of at least one obesity‐associated obstetric or perinatal complications (one or more of GDM, pre‐eclampsia, pregnancy‐induced hypertension, preterm birth, post‐partum haemorrhage, SGA, LGA and asphyxia) was 1.49 times higher (95% CI 1.03–2.15) in women with metabolically unhealthy versus healthy obesity.^32^ As they are related to cardiometabolic markers in the EOSS, we included GDM, PET and PIH in the scoring criteria rather than assessing them as pregnancy outcomes associated with EOSS. According to their criteria, 181 (33.1%) were classified as metabolically unhealthy. This contrasts with the present study, in which 81.9% had raised EOSS scores in early pregnancy. The higher proportion of women with metabolically unhealthy obesity in our study may explain the limited predictive value found. Of note, the factors considered in the metabolic profiling differed from that of the EOSS as there were only four criteria, none of which included cholesterol. Flanagan et al. grouped women with obesity based on their metabolic profiling and compared infant anthropometric measures. Although the sample size was small, including only 13 women in the metabolically healthy group and nine in the metabolically unhealthy group, they found higher birthweight and infant adiposity in the metabolically unhealthy group.^33^ They classified women as metabolically unhealthy if they had two or more of the following, systolic blood pressure >130 mmHg or diastolic blood pressure >85 mmHg, HDL cholesterol <50 mg/dl, LDL cholesterol ≥100 mg/dl, triglycerides ≥150 mg/dl, and glucose ≥100 mg/dl. The metabolically healthy group had none of these factors. While this study had more scoring criteria including LDL and HDL cholesterol, it did not include total cholesterol in their scoring.^33^

We found that the main driver of higher EOSS score was total cholesterol and its' inclusion in the scoring system may have limited its' potential in stratifying risk of adverse outcomes. Potter and Nestel found cholesterol levels increased by 50% throughout gestation.^34^ Given the high levels of cholesterol expected during pregnancy, future work could consider the use of alternative scoring tools, such as varying criteria that do not include total cholesterol. This includes the criteria for the metabolic syndrome or the cardiometabolic disease staging system.^10^, ^28^, ^35^ This is further supported by evidence suggesting that high triglycerides and low‐HDL increase LGA.^36^ Although others have found that pre‐pregnancy BMI and gestational weight gain are greater drivers of this relationship compared to cardiometabolic profiles.^37^

Most women experience cardiometabolic change throughout gestation, including increased total cholesterol, LDL cholesterol and triglycerides and reduced HDL cholesterol.^38^ Some data suggest that increases in cholesterol occur from the second trimester onwards while others have found higher median cholesterol in all trimesters compared to pre‐pregnancy values.^34^, ^39^, ^40^ In early pregnancy, 81.9% of women in our study had some degree of dyslipidaemia. This increased to 98.9% in late pregnancy. Although increases in cardiometabolic factors are expected in pregnancy, reaching high values may increase the risk of post‐partum dyslipidaemia. In women with a history of GDM, LDL cholesterol levels >3.56 mmol/L in the second trimester were associated with increased risk of dyslipidaemia at 6–12 weeks post‐delivery.^41^ Darmady and Postle found cholesterol levels remained elevated up to 36 weeks post‐partum.^39^ The degree of change experienced may also influence associations with outcomes. In a prospective study of 575 women, Bever et al. found a 10 mg/dL increase in triglycerides from preconception to 28 weeks was associated with increased odds of LGA and a 10 mg/dL decrease was associated with reduced odds of SGA and LGA.^42^ On this basis, more research on the extent of cardiometabolic changes experienced in pregnancy is warranted.

A recent systematic found that a low glycaemic index/load dietary pattern is associated with reduced fasting glucose, LDL cholesterol, Apo B triglycerides and systolic blood pressure, in adults with type two diabetes and a raised BMI.^43^ Cha et al. found that adolescents with higher modified‐EOSS scores reported lower diet quality that those with lower risk factors. In addition, those with a modified‐EOSS score of zero reported greater percentage energy from protein consumption.^44^, ^45^ While previous studies found the PEARS intervention group had lower dietary glycaemic load and higher protein intake compared to the control group, we did not find a difference in EOSS scores in early to late pregnancy based on the study group.^18^, ^46^ Interventions to promote a more favourable cardiometabolic profile in women during preconception may therefore prove more efficacious in ensuring healthier levels during pregnancy and post‐partum.^47^, ^48^, ^49^, ^50^

### 4.3 Strengths and limitations

Our study takes a novel and pragmatic approach to exploring the cardiometabolic changes experienced in pregnancy by tracking women against clinically relevant cut‐offs for dyslipidaemia, rather than comparing raw values. Pregnancy‐specific cut‐offs for cardiometabolic parameters have not yet been developed.^15^, ^16^ The American Heart Association, the American College of Cardiology and others advocate for the use of sex‐specific considerations in cardiovascular risk assessments.^51^ A relevant core outcome set is the Core Outcome Set for Studies on Obesity in Pregnant Patients, and this project highlighted a need for greater measurement of outcomes relating to emotional functioning.^52^, ^53^, ^54^, ^55^ Use of a validated and internationally relevant questionnaire to assess well‐being allows for meaningful comparison with the literature and addresses gaps in EOSS studies and pregnancy obesity that did not consider mental health.^13^ Use of the Benjamini‐Hochberg adjustment for multiple comparisons adds statistical rigour to the data.^56^ This study is not without limitations. Selection of women with sufficient criteria for EOSS classification may introduce selection bias. We did not have sufficient data to allow for application of the functional aspect of EOSS, or application of stage 3 or 4 EOSS. Other studies in non‐pregnant individuals have used functional limitation and activities of daily living in the functional assessment but interpretation of this may be difficult in pregnancy due to the associated functional decline throughout gestation, especially for those with obesity.^13^, ^57^, ^58^ This was an exploratory analysis of data collected as part of a randomized controlled trial, and it is possible that our study did not have statistical power to find differences in pregnancy outcomes. Regardless, the spread of EOSS stages in our cohort does not support application in its current format for the purpose of treatment prioritization.

## 5 CONCLUSION

Most women with overweight or obesity have raised EOSS scores in early pregnancy, and EOSS score did not predict pregnancy outcomes. Efforts to improve cardiometabolic health in women with BMI ≥25 kg/m^2^ before pregnancy are warranted.

## CONFLICT OF INTEREST

No conflict of interest was declared.

## AUTHOR CONTRIBUTIONS

*Conceptualization*: Sarah Louise Killeen and Fionnuala M. McAuliffe; *methodology*: Sarah Louise Killeen, Aisling A. Geraghty, Cara A. Yelverton, Shane Eakins, Lily Farrell and Jillian F. Fagan; *software*: John Mehegan; *validation*: Sarah Louise Killeen and John Mehegan; *formal analysis*: Sarah Louise Killeen; *investigation*: Maria A. Kennelly and Fionnuala M. McAuliffe; *resources*: Fionnuala M. McAuliffe; *data curation*: Sarah Louise Killeen, John Mehegan and Fionnuala M. McAuliffe; *writing – original draft preparation*: Sarah Louise Killeen; *writing – review and editing*: Sarah Louise Killeen, Cara A. Yelverton, Aisling A. Geraghty, Maria A. Kennelly, Shane Eakins, Lily Farrell, Jillian F. Fagan, John Mehegan and Fionnuala M. McAuliffe; *visualization*: Sarah Louise Killeen, Aisling A. Geraghty and Fionnuala M. McAuliffe; *supervision*: Fionnuala M. McAuliffe; *project administration*: Sarah Louise Killeen, Maria A. Kennelly and Fionnuala M. McAuliffe; *funding acquisition*: Fionnuala M. McAuliffe. All authors have read and agreed to the published version of the manuscript.
